# Biochemical and molecular characterization of fungal isolates from California annual grassland soil

**DOI:** 10.1186/s13068-025-02651-4

**Published:** 2025-05-31

**Authors:** Taren Bouwman, Lauren Higa, Caitlyn Lee, Shaina Young, Arel Ragasa, Gregory Bonito, Nhu H. Nguyen, Zhi-Yan Du

**Affiliations:** 1 Department of Molecular Biosciences and Bioengineering, University of Hawaiʻi at Mānoa, Honolulu, HI 96822 USA; 2 Department of Plant, Soil and Microbial Sciences, Michigan State University, East Lansing, MI 48824 USA; 3 Department of Tropical Plant and Soil Science, University of Hawaiʻi at Mānoa, Honolulu, HI 96822 USA

**Keywords:** Fungi, Biodiversity, Biofuel, Lipids, Nutraceuticals

## Abstract

Fungi play a pivotal role in ecosystem functionality, driving processes such as decomposition, nutrient cycling, and symbiotic interactions. Their wide enzymatic strategies enable the breakdown of complex organic materials and the valorization of organic waste streams, providing sustainable pathways for bioproduct development. Fungi also exhibit significant potential in industrial applications, particularly in biofuel and nutraceutical production, owing to their high lipid content and adaptability to diverse feedstocks. Genera such as *Aspergillus*, *Mortierella*, and *Linnemannia* have demonstrated exceptional lipid production capabilities and unique fatty acid profiles, including high yields of nutraceuticals like arachidonic acid (ARA) and oleic acid. This study explored uncharacterized fungal strains isolated from California grassland soils, analyzing their phylogeny, morphology, growth rates, lipid content, and fatty acid profiles. Results revealed notable genetic and physiological diversity among the isolates, with *Mortierella* strains emerging as the most promising for industrial applications due to their superior lipid content and productivity of ARA and oleic acid. Confocal microscopy confirmed consistent lipid droplet morphology, while phylogenetic analysis uncovered novel species-level diversity. Key strains were identified for biofuel and nutraceutical production, highlighting their industrial potential. These findings underscore the versatility of fungi as biotechnological tools and provide a foundation for further exploration and utilization of these promising strains in industrial processes.

**Graphical Abstract:**

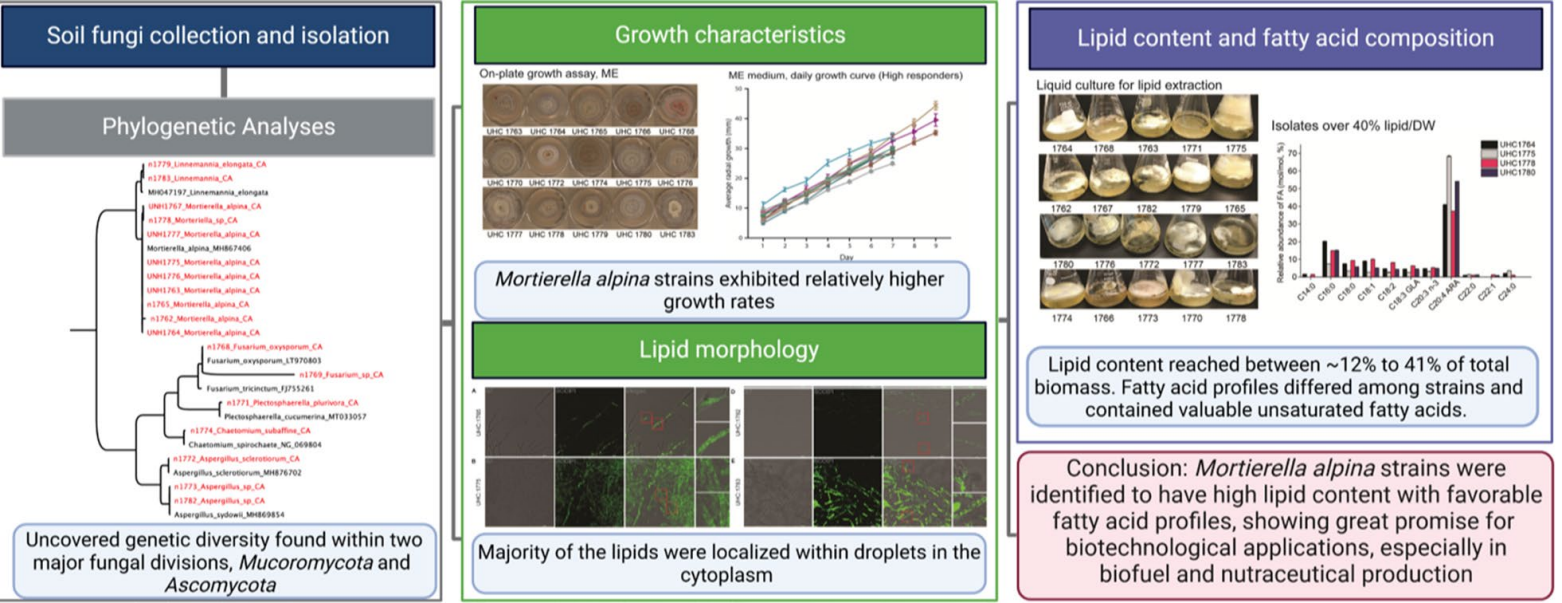

## Introduction

Fungi play indispensable roles in ecosystem functionality, primarily through their contributions to decomposition, nutrient cycling, and symbiotic relationships. As primary decomposers, fungi break down complex organic matter, recycling nutrients back into the environment. Their extensive mycelial networks and sophisticated enzymatic systems enable efficient decomposition of complex biological materials such as lignin and cellulose [1]. These enzymatic capabilities also allow fungi to utilize organic waste streams, converting waste into valuable bioproducts [2]. Employing fungi for waste valorization not only reduces production costs, but also mitigates the climate impact of carbon-intensive waste processing systems by diverting organic waste from landfills or incineration. 

Fungi have diverse industrial applications, particularly in addressing the growing demand for biofuels and nutraceuticals. Their high lipid content and ability to utilize a wide range of feedstocks highlight their potential as biofuel production systems [2]. Additionally, fungi are a promising source of nutraceuticals such as arachidonic acid (ARA), oleic acid, and eicosapentaenoic acid (EPA). ARA is an essential fatty acid crucial for cellular function, while oleic acid and EPA have been shown to reduce inflammation and promote cardiovascular health [3–5].

Fungi are vital producers of industrially significant biomolecules, including enzymes, secondary metabolites, and lipids. Fungal enzymes are essential in industries like paper production, where *Aspergillus niger* is used to produce xylanase for bio-bleaching paper pulp [6]. Secondary metabolites from fungi have been pivotal in drug discovery, exemplified by the cytotoxic versixanthones produced by *Aspergillus versicolor* on human cancer cell lines [7]. Additionally, *Aspergillus* sp. EM2018 has been shown to produce over 25.2% lipid content per dry biomass, underscoring its potential for biofuel production [8]. *Aspergillus flavus* NDA04a has also demonstrated the ability to catalyze triglyceride transesterification for biodiesel production [9].

While *Aspergillus* is extensively studied, other genera, such as *Mortierella*, exhibit enhanced production capabilities for specific applications. For instance, *Mortierella isabellina* produces 47.9% lipid content per dry biomass, highlighting it for biofuel production [10]. Similarly, *Mortierella alpina* has been found to produce high amounts of ARA, constituting 41.1% of its total fatty acids, making it valuable for nutraceutical production [11]. Additionally, *M. alpina* effectively flocculated *Chlamydomonas reinhardtii*, providing a biological method for algae harvesting without microfilters [12].

Less extensively studied genera, such as *Linnemannia*, also show promise. *Linnemannia* species produce significant amounts of oleic acid and EPA, while also forming symbiotic relationships with plants such as *Arabidopsis*, stimulating aerial root growth. Moreover, *Linnemannia* species have demonstrated innovative biofuel production systems in conjunction with algae. For example, *Linnemannia elongata* (= *Mortierella elongata*) cultivated with *Nannochloropsis oceanica* achieved greater oil productivity than either species cultivated individually [13, 14]. The genus has also been noted to host bacterial endosymbionts, suggesting potential for novel industrial applications [15].

While significant progress has been made in characterizing fungal strains for lipid production, further exploration of uncharacterized strains is essential to fully evaluate their potential for industrial applications.

Fungi also form symbiotic relationships with various organisms, including plants, algae, and bacteria, facilitating nutrient exchange and providing protection [16, 17]. For example, under specific conditions, fungi and algae can form photosynthetic mycelium, exchanging nutrients to enhance the fitness of both species [13]. Additionally, fungi form endosymbiotic relationships with other microorganisms, such as *Mycoavidus cysteinexigens*, which further expands their ecological and industrial relevance as symbiotic production systems [18].

In this study, we evaluated a variety of uncharacterized fungal strains isolated from California by assessing their phylogeny, morphology, growth rates, total lipid contents, and fatty acid profiles. We isolated and cultivated these fungi, determined their growth rates, and analyzed their total lipid content. Lipid profiles were characterized using gas chromatography, while phylogenetic relationships were established through ITS and LSU sequencing. Imaging of mycelial networks further elucidated morphological characteristics. Through these analyses, we identified novel strains optimized for rapid growth, high lipid content, and enhanced ARA production. Three genera of fungi were determined through these analyses: *Linnemania*, *Aspergillus*, and *Mortierella*. Of these, *Mortierella* had the highest diversity and highest lipid productivity rates. Five unique strains among these genera were highlighted for their overall lipid production rates, ARA production rate, and oleic acid production rate.

## Results and discussion

### Phylogeny and genetic identification of isolates

**Fig. 1:**
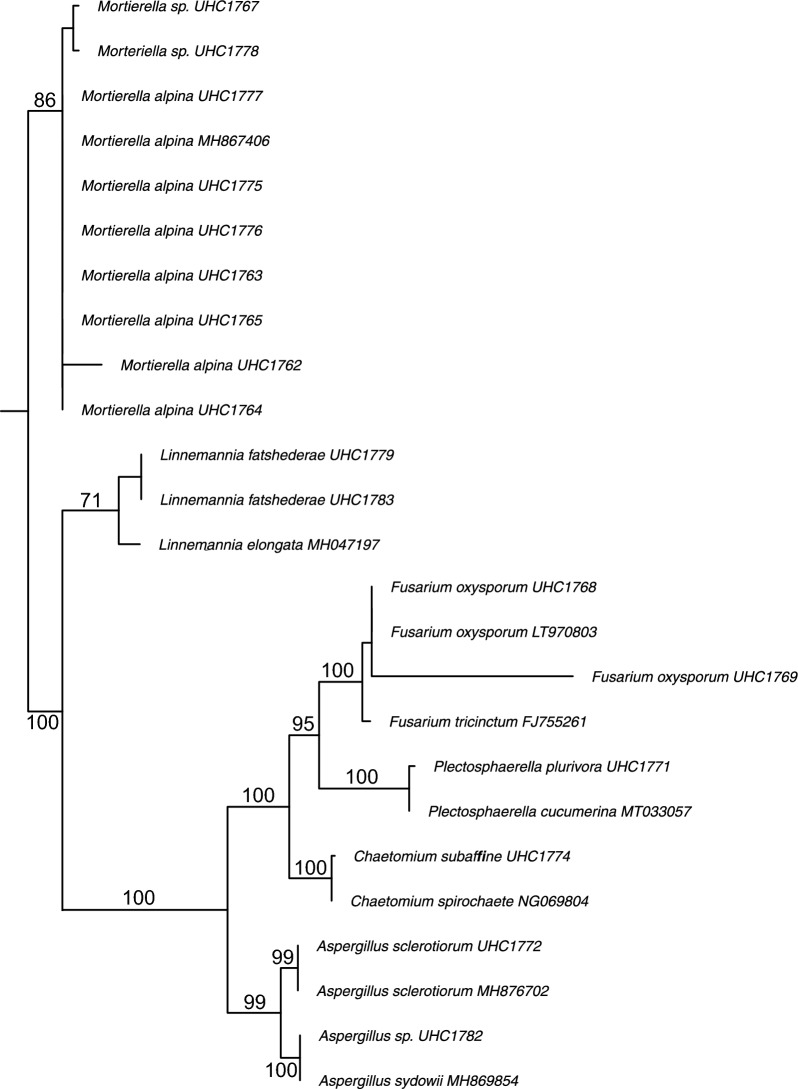
Phylogenetic tree of selected fungal strains based on Sanger sequencing of the internal transcribed spacer (ITS) and large ribosomal subunit (LSU) rRNA gene. Sequences from known species were included to enhance the resolution of the tree. Numbers at the nodes are maximum likelihood bootstrap values

The fungal strains were isolated from California annual grassland soils and were subjected to Sanger sequencing of the internal transcribed spacer (ITS) and large ribosomal subunit (LSU) rRNA genes to determine their taxonomic classification at the genus and species levels. The resulting sequences, along with reference sequences from related strains, were used to construct a phylogenetic tree. This analysis revealed the presence of two major fungal phyla: *Mucoromycota* and *Ascomycota* (Fig. 1).

The two *Mucoromycota* genera present in this study were *Mortierella* and *Linnemannia*. The vast majority of *Mortierella* were *M. alpina*, apart from two species of *Mortierella,* which were unidentified species. These two unidentified *Mortierella* species were closely related and had moderate ML support (Fig. 1). The only *Linnemannia* species found were *Linnemannia fatschederae*, a relative to *Linnemannia elongata*, with moderate ML support (Fig. 1). 

Four Ascomycota were found in this study: *Aspergillus*, *Chaetomium*, *Fusarium*, and *Plectosphaerella.* Within these genera, there was less species variability compared to the wide range of *Mortierella* identified. The *Aspergillus sclerotiorum* isolate was highly similar to the reference sequence, while the unidentified *Aspergillus* sp. was related to *Aspergillus sydowii*. The *Chaetomium subaffine* isolate was highly similar to the reference *C. spirochete*, while the *Plectosphaerella plurivora* isolate was closely related to the reference *Plectosphaerella cucumerina*. Lastly, the two *Fusarium* species identified as *F. oxysporum* and *F. tricinctum* were highly similar to their respective reference species, although they likely belong to species complexes which can only be identified further by sequencing of the *TEF1-α* gene*.* All *Ascomycota* isolates from this study had good ML support (Fig. 1).

### Lipid morphology is consistent across samples

**Fig. 2:**
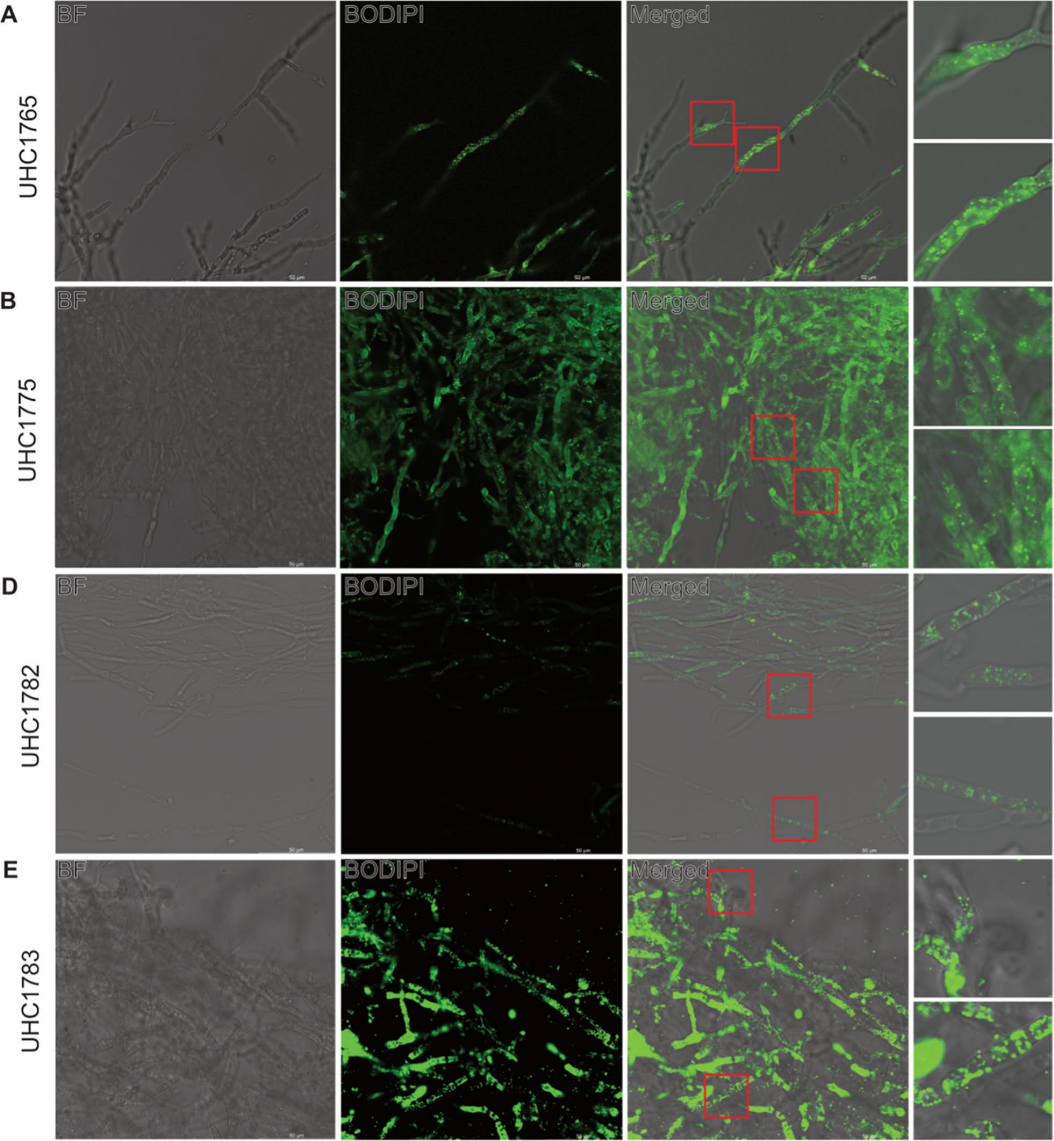
Confocal microscopy photographed at 63 × of selected fungal strains with bright-field, BODIPI, and merged images. Merged images include two zoomed in representative areas. BODIPI images demonstrate localization of oil deposits within mycelium

To investigate the lipid morphology of the fungal strains, confocal microscopy was performed using BODIPY staining, identifying non-polar regions to determine lipid localization. The analysis revealed that the majority of lipids for the strains imaged were localized within droplets in the cytoplasm (Fig. 2). Notably, *M. alpina* strains exhibited significantly higher lipid content compared to other strains, as detailed in Table 1 (*Mortierella* vs. non-*Mortierella*). Despite this higher lipid content, there was no visual evidence of increased lipid droplet size or droplet density in *M. alpina* compared to other strains.

**Table 1:** Comparison of total lipid content of all *Mortierella* fungal isolates vs all other non-*Mortierella* fungal isolates

Paired samples *t*-test					
Measure 1		Measure 2	*t*	*df*	*p*
*Mortierella*	–	Not *Mortierella*	4.404	7	0.003

Student's *t*-test

The morphological differences between these strains were also evident. *M. alpina* strains UHC1765 and UHC1775 displayed additional BODIPY signal along the border of the mycelium, a feature not observed in *Linnemannia* strains UHC1782 and UHC1783 (Fig. 2A, B). This staining difference could also indicate the presence of additional lipids localized at the mycelial border, potentially explaining the total lipid content difference between *Mortierella* and other strains (Table 1). Although BODIPY staining should not be utilized as a quantitative metric, this staining pattern does deserve further investigation.

### Growth assays using two different media

**Fig. 3:**
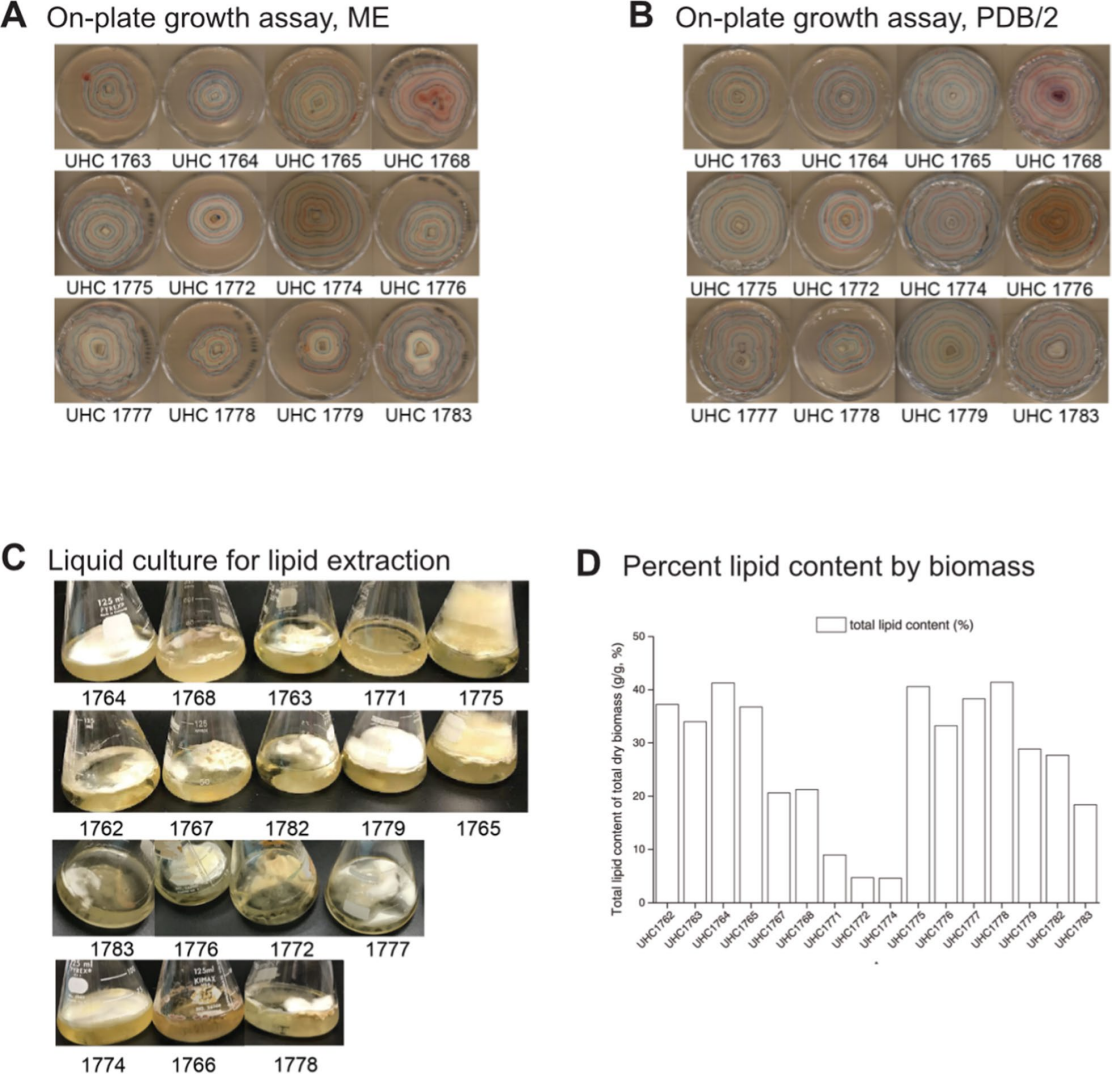
Images of some of the plates utilized to quantify growth rates for ME (**A**) and PDB/2 (**B**) based media. A selection of the ME liquid culture utilized for lipid extractions and quantification (**C**) and the resulting percent lipid content by total biomass (**D**)

In order to quantify the growth rates of these isolates, they were cultivated on Malt Extract media (ME) and Potato Dextrose Broth media (PDB/2)-based plates, and the average daily growth was recorded. Many strains showed similar growth rates, while 2 strains grew significantly faster on PDB/2 than ME media: UHC1779, *L. fatshederae*, and UHC1782, *Aspergillus sp.* (Table 2). These trends are further illustrated with images from the growth plate assays. While most stains appear similar on both media types, UHC1779 grows to a larger radius on PDB/2 than on ME (Fig. 3A, B).

**Table 2:** Student *t*-test comparing fungal isolate growth rates grown on ME or PDB/2 media based agarose plates

Paired samples *t*-test					
Measure 1		Measure 2	*t*	*df*	*p*
1762 ME	–	1762 PDB/2	0.786	6	0.462
1763 ME	–	1763 PDB/2	0.323	7	0.756
1764 ME	–	1764 PDB/2	− 1.559	7	0.163
1765 ME	–	1765 PDB/2	− 1.157	7	0.285
1767 ME	–	1767 PDB/2	− 1.504	7	0.176
1768 ME	–	1768 PDB/2	− 0.789	9	0.450
1771 ME	–	1771 PDB/2	− 1.211	6	0.271
1772 ME	–	1772 PDB/2	− 0.588	6	0.578
1774 ME	–	1774 PDB/2	− 0.203	9	0.844
1775 ME	–	1775 PDB/2	− 2.311	7	0.054
1776 ME	–	1776 PDB/2	− 1.966	8	0.085
1777 ME	–	1777 PDB/2	1.945	7	0.093
1778 ME	–	1778 PDB/2	0.907	7	0.395
1779 ME	–	1779 PDB/2	− 3.364	8	0.010
1782 ME	–	1782 PDB/2	− 3.124	7	0.017
1783 ME	–	1783 PDB/2	− 1.564	7	0.162

### Growth rates and lipid composition of notable isolates

#### *Aspergillus*

**Fig. 4:**
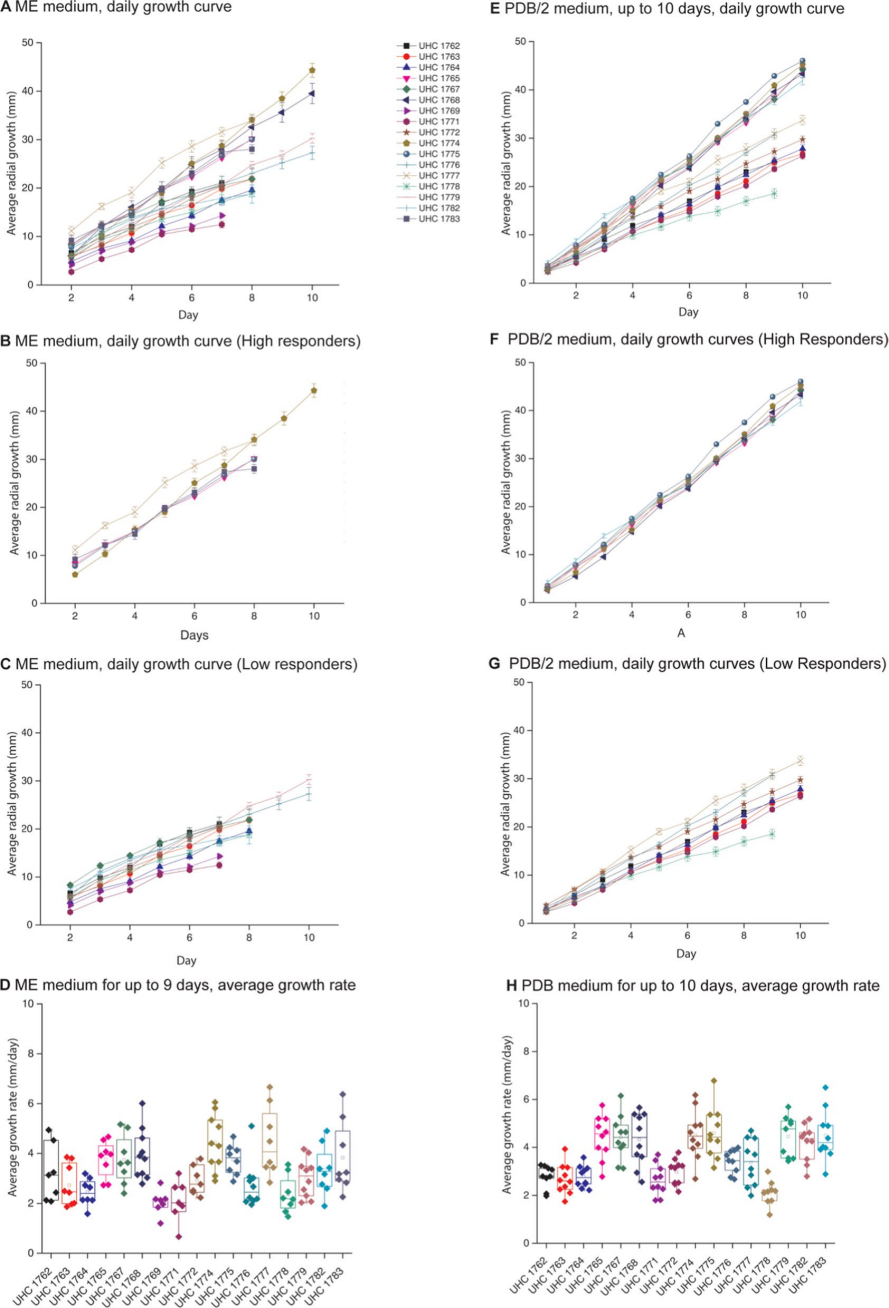
Growth curves for fungal isolates grown on ME (**A**–**D**) or PDB/2 (**E**–**H**) media. ME growth rates combined (**A**) separated by high responders above 24 mm after 7 days of growth (**B**) and low responders bellow 26 mm growth after 7 days (**C**). PDB/2 growth rates combined (**E**) separated by high responders above 26 mm after 7 days of growth (**F**) and low responders bellow 26 mm growth after 7 days (**G**). Overall average growth rate of each fungal isolate for ME plates (**D**) and PDB/2 plates (**H**)

The *Aspergillus* strains (UHC1772, UHC1782) exhibited no significant differences in growth when cultured on ME media. However, a significant difference was observed when the strains were grown on PDB/2 media, with *Aspergillus sclerotiorum* strain UHC1772 showing slower growth compared to *Aspergillus* sp. strain UHC1782 (Fig. 4D, H). This difference in growth could be due to the wide variability in *Aspergillus* taxonomy [19].

Further examination of the growth patterns in liquid media revealed distinct morphological differences. Strain UHC1772 grew primarily on the surface of the liquid, while strain UHC1782 formed a three-dimensional structure extending above the liquid surface (Fig. 3C). This morphological variation corresponds with differences in lipid content, with strain UHC1772 has lipid content below 12% of total biomass, in contrast to strain UHC1782, which exhibited a lipid content exceeding 25% (Fig. 3D). These lipid content patterns are consistent with previous research on lipid variability in *Aspergillus* [20]

**Fig. 5:**
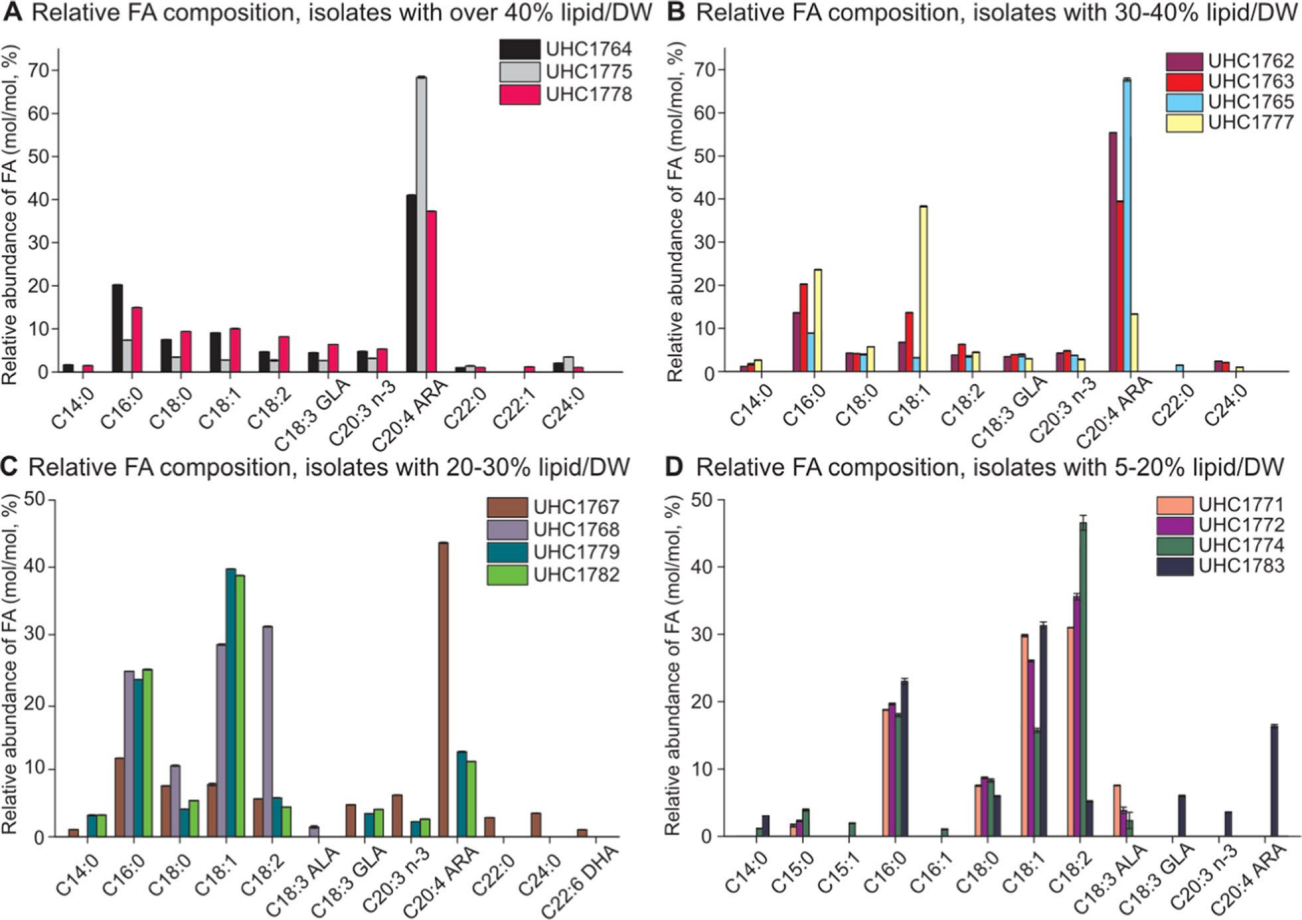
Fatty acid profiles of fugal isolates separated by above 40% (**A**), 30–40% (**B**), 20–30% (**C**), and 5–20% (**D**) lipid mass by total mass

The differences between these strains extended to their lipid profiles as well. Strain UHC1772 was primarily composed of linoleic acid (C18:2), constituting 35% of its lipid content, whereas strain 1782 contained only 5% linoleic acid (Fig. 5C, D). The lower linoleic acid content in strain 1782 was compensated by higher levels of other fatty acids, including palmitic acid (C16:0), oleic acid (C18:1), and arachidonic acid (C20:4 ARA) (Fig. 5C, D).

These findings highlight significant physiological and biochemical differences among the *Aspergillus* strains, particularly between the known species *A. sclerotiorum* (UHC1772) and the unknown *Aspergillus* species: UHC1782. The variations in growth behavior, lipid content, and fatty acid composition suggest that these strains may have different metabolic pathways and ecological adaptations, which could be further explored to understand their potential applications in biotechnology and industrial processes. The unique characteristics of strain *Aspergillus* sp. UHC1782, especially its higher lipid content and distinct fatty acid profile, make it a particularly interesting candidate for further study.

#### *Linnemannia*

When examining the *Linnemannia* species, no significant differences in growth rates were observed between either ME or PDB/2 media (Fig. 4C, D). However, notable differences in morphology were apparent when these species were grown in liquid culture. *Linnemannia fatshederae*, strain UHC1779 developed a large three-dimensional structure extending above the liquid surface, while the *L. fatshederae*, strain UHC1783, primarily grew as a surface layer on the liquid (Fig. 3C). Previous research shows that *Mortierella* species, now classified as *Linnemannia* possess, morphological variability [14, 21].

This morphological difference was accompanied by variations in lipid content. *L. fatshederae*, UHC1779, had approximately 30% total lipid content, higher than the other *L. fatshederae*, UHC1783, which only had 20% total lipid content (Fig. 5D). The fatty acid composition also showed slight differences, with *L. elongata* containing about 10% more oleic acid (C18:1) and 10% less arachidonic acid (C20:4 ARA) than the unidentified species.

These findings suggest that while the growth rates of these *Linnemannia* species are similar when cultivated on solid media, their morphological adaptations in liquid culture and differences in lipid content and composition could reflect distinct ecological strategies or metabolic capabilities. The higher lipid content and unique fatty acid profile of *L. fatshederae strain*: UHC 1779, particularly its increased oleic acid levels, may have implications for its potential industrial applications in lipid production. Further research is warranted to explore these differences and their underlying causes.

#### *Mortierella*

In this study, nine different *Mortierella* strains were isolated, demonstrating some of the highest growth rates and total lipid contents among all the fungal strains analyzed. The growth rates of *Mortierella* strains varied widely, with strain UHC1778 showing one of the lowest growth rates while strain UHC1777 exhibited the highest (Fig. 4). The differences in growth rates were statistically significant, with strain UHC1778 growing significantly slower than strains UHC1765, UHC1767, UHC1775, and UHC1777 (Table 3). Strain UHC1777 had a significantly higher growth rate compared to strains UHC1762, UHC1763, UHC1764, UHC1776, and UHC1778 (Table 3). The variability of *Mortierella* in this study is consistent with previous research on *Mortierella* [22].

**Table 3:** Student *t*-test of *Mortierella* isolates, determining significant differences in growth rate

Paired samples *t*-test				
Measure 1	Measure 2	*t*	*df*	*p*
1762 ME	1763 ME	1.204	6	0.274
1762 ME	1764 ME	1.940	6	0.100
1762 ME	1765 ME	− 1.788	6	0.124
1762 ME	1767 ME	− 1.156	6	0.292
1762 ME	1775 ME	− 1.925	6	0.103
1762 ME	1776 ME	0.396	6	0.706
1762 ME	1777 ME	− 4.677	6	0.003
1762 ME	1778 ME	1.742	6	0.132
1763 ME	1764 ME	1.353	7	0.218
1763 ME	1765 ME	− 4.836	7	0.002
1763 ME	1767 ME	− 3.729	7	0.007
1763 ME	1775 ME	− 4.942	7	0.002
1763 ME	1776 ME	− 0.281	7	0.787
1763 ME	1777 ME	− 4.615	7	0.002
1763 ME	1778 ME	1.215	7	0.264
1764 ME	1765 ME	− 7.555	7	< 0.001
1764 ME	1767 ME	− 5.726	7	< 0.001
1764 ME	1775 ME	− 12.490	7	< 0.001
1764 ME	1776 ME	− 1.067	7	0.322
1764 ME	1777 ME	− 4.804	7	0.002
1764 ME	1778 ME	0.357	7	0.731
1765 ME	1767 ME	0.140	7	0.893
1765 ME	1775 ME	0.133	7	0.898
1765 ME	1776 ME	2.060	7	0.078
1765 ME	1777 ME	− 2.231	7	0.061
1765 ME	1778 ME	6.029	7	< 0.001
1767 ME	1775 ME	− 0.054	7	0.959
1767 ME	1776 ME	1.777	7	0.119
1767 ME	1777 ME	− 1.648	7	0.143
1767 ME	1778 ME	4.634	7	0.002
1775 ME	1776 ME	2.267	7	0.058
1775 ME	1777 ME	− 2.103	7	0.073
1775 ME	1778 ME	6.470	7	< 0.001
1776 ME	1777 ME	− 2.908	7	0.023
1776 ME	1778 ME	2.089	7	0.075
1777 ME	1778 ME	5.813	7	< 0.001

The *Mortierella* strains also displayed differences in lipid content, morphology, and composition. When grown in liquid culture, two distinct growth patterns were observed: strains UHC1763, UHC1764, UHC1767, UHC1776, UHC1777, and UHC1778 grew on the surface of the liquid, while strains UHC1765 and UHC1775 extended their growth out of the flask. Most of these isolates had a lipid content ranging from 35 to 45% of their biomass, with the exception of strain UHC1767, which had a notably lower lipid content of 20% (Fig. 3D).

The lipid composition among the *Mortierella* strains was generally consistent, with arachidonic acid (C20:4 ARA) being the predominant fatty acid, comprising 40 to 70% of the total lipids. This was followed by palmitic acid (C16:0), which accounted for 7 to 20% of the lipid content (Fig. 5). However, strain UHC1777 was an exception, with only 15% ARA and a much higher proportion of oleic acid (C18:1), nearly 40%, whereas other *Mortierella* strains contained no more than 15% oleic acid (Fig. 5B).

These findings highlight the diversity within *M. alpina* strains in terms of growth behavior, lipid content, and fatty acid composition. *M. alpina*, in particular, emerged as the most optimal strain for arachidonic acid and overall fatty acid production, based on its high growth rates, substantial lipid content, and elevated ARA levels. 

### Relative lipid production of isolates

The analysis of relative accumulation rates of specific fatty acids clearly indicates that the *M. alpina* strains are the highest-performing species in terms of lipid accumulation rate (Table 4). While this finding provides valuable insights into the lipid production rates of the fungal isolates, it is important to note that these calculations assume consistent hyphal density across all strains of the species. Looking past this limitation, *M. alpina* strains consistently outperformed others in lipid production, with specific strains being optimized for the production of particular fatty acids.

**Table 4:** Relative lipid accumulation rates of fungal isolates cultivated with ME media

Strain	Growth rate	Percent lipid by biomass	Relative Lipid accumulation rate	16:0	18:0	18:1	18:2	20:4 ARA	Relative 16:0 accumulation rate	Relative 18:0 accumulation rate	Relative 18:1 accumulation rate	Relative 18:2 accumulation rate	Relative 20:4 ARA accumulation rate
1762	3.211	37.0	11.98	13.0%	4.0%	**8.0%**	4.0%	55.0%	1.557	0.479	0.958	0.479	6.588
1763	2.733	34.0	7.97	20.0%	4.0%	**13.0%**	6.0%	40.0%	1.594	0.319	1.036	0.478	3.189
1764	2.448	41.0	7.71	20.0%	8.0%	**9.0%**	5.0%	41.0%	1.543	0.617	0.694	0.386	3.162
1765	3.773	41.0	18.33	9.0%	4.0%	**2.0%**	4.0%	68.0%	1.649	0.733	0.367	0.733	12.462
1767	3.741	20.0	8.79	11.0%	8.0%	8.0%	**6.0%**	44.0%	0.967	0.703	0.703	0.527	3.868
1768	3.952	21.0	10.30	25.0%	10.0%	29.0%	**31.0%**	0.0%	2.574	1.030	2.986	3.192	0.000
1771	2.105	10.0	1.39	19.0%	8.0%	**30.0%**	31.0%	0.0%	0.264	0.111	0.417	0.431	0.000
1772	2.915	5.0	1.33	20.0%	7.0%	**27.0%**	36.0%	0.0%	0.267	0.093	0.360	0.480	0.000
1774	4.430	5.0	3.08	17.0%	8.0%	**16.0%**	48.0%	0.0%	0.524	0.247	0.493	1.479	0.000
1775	3.756	41.0	18.16	7.0%	4.0%	**3.0%**	3.0%	69.0%	1.271	0.726	0.545	0.545	12.530
1776	2.728	33.0	7.71	10.0%	4.0%	**3.0%**	3.0%	62.0%	0.771	0.308	0.231	0.231	4.782
1777	4.464	37.0	23.16	14.0%	5.0%	**39.0%**	5.0%	13.0%	3.242	1.158	9.031	1.158	3.010
1778	2.356	41.0	7.14	15.0%	9.0%	**10.0%**	7.0%	38.0%	1.071	0.643	0.714	0.500	2.714
1779	3.026	34.0	9.77	24.0%	4.0%	**40.0%**	**6.0%**	12.0%	2.346	0.391	3.910	0.586	1.173
1782	3.330	33.0	11.49	25.0%	5.0%	39.0%	5.0%	11.0%	2.873	0.575	4.481	0.575	1.264
1783	3.828	19.0	8.74	23.0%	6.0%	31.0%	5.0%	17.0%	2.010	0.524	2.710	0.437	1.486

Combining results from Figs. 2D, 3D, and 4. Growth rate is derived from radial growth measured on ME plates and assuming consistent density for all strains

Four *M. alpina* strains stood out for their overall lipid production and specific fatty acid profiles: UHC1765, UHC1768, UHC1775, and UHC1777. Among these, strain UHC1777 exhibited the highest overall relative lipid accumulation rate and excelled in the accumulation of palmitic acid (16:0), stearic acid (18:0), and oleic acid (18:1) (Table 2 calculations). Strains UHC1765 and UHC1775 also demonstrated high overall relative lipid accumulation rates, particularly excelling in the production of arachidonic acid (20:4 ARA), having the highest overall ARA accumulation rate (Table 4). Strain UHC1768, although below average in overall lipid accumulation, had the highest accumulation rate of linoleic acid (18:2) and thus is relevant for industrial use (Table 4).

Based on this analysis, the *M. alpina* strain UHC1777 emerges as the most promising variant for overall lipid production, particularly for applications such as biofuel production. Additionally, *M. alpina* strains UHC1765 and UHC1775, with their similar high rates of ARA accumulation, present optimal use cases for the production of arachidonic acid, which has various industrial applications.

## Conclusion

This study presents a detailed analysis of fungal strains isolated from California grassland soils, with a focus on their phylogeny, growth characteristics, lipid content, and fatty acid composition. The phylogenetic analysis identified two major fungal phyla, *Mucoromycota* and *Ascomycota*, and uncovered significant genetic diversity within these groups, suggesting the presence of closely related but distinct species. Among the isolates, *M. alpina* strains stood out, exhibiting some of the highest growth rates and total lipid contents. This finding is consistent with previous examinations into filamentous fungi for biofuel potential [23]. In combination, these two traits are important for the highest rate of total lipid production and specific lipid production, highlighting their potential for industrial biofuel and nutraceutical production applications.

Confocal microscopy revealed consistent lipid droplet morphology concentrated within the cytoplasm across all strains. Notably, the staining patterns along the mycelial borders in *Mortierella* strains suggest the presence of additional lipids, which could be further explored in future studies.

The findings of this study underscore the variability in growth rates and lipid compositions, even among strains of the same species. *M. alpina* strains, in particular, show great promise for biotechnological applications, especially in biofuel and nutraceutical production. The high lipid content and specific fatty acid profiles of these strains, coupled with their unique growth characteristics, emphasize the importance of careful strain selection and optimization for industrial use. Specifically, strain UHC1777 emerges as a strong candidate for overall lipid production, while strains UHC1765 and UHC1775 are optimal for arachidonic acid (ARA) production, and strain UHC1768 shows potential for linoleic acid production. These strains meet or exceed previously reported lipid production levels for *Mortierella* [23], [24].

Further research into these strains lipid production capabilities, either when forming symbioses with algae and other microorganisms or when organic waste is utilized as a nutritional source, will further reveal specific industrial uses for these strains and the foundational understanding of new industrial applications for filamentous fungi.

## Methods

Fungi were isolated from grassland soils, dominated by *Avena* sp. collected at the University of California Hopland Research and Extension Center (HREC). 1 g of soil was mixed with 1 mL of sterile water, diluted to 10^–5^, plated on ME solid media plates (15 g malt extract, 2.5 g peptone, 10 g agar, 1 L water) and incubated at 25° C. Colonies were serially isolated until a pure culture was obtained. Cultures were maintained on ME solid medium following a published protocol [25].

### BODIPI staining and confocal imaging

To stain and image lipids within mycelium, a portion of the mycelium cultivated on a PDB/2 growth plate (12 g potato dextrose broth, 0.5 g yeast extract, 10 g agar, 1 L water, as reported previously) [26] was transferred to a 1.5-ml tube containing 1.0 ml liquid PDB/2 (12 g potato dextrose broth, 0.5 g yeast extract, 1 L water) and allowed to recover overnight. Following the published protocol [26], 1 μl of 5 mg/ml BODIPI (Thermo Fisher Scientific, cat# D3921, Waltham, MA, USA) was added to the 1.5-ml tube and incubated for 20 min on a rolling incubator Roto-Therm mini plus by Benchmark in the dark at room temperature. Liquid was removed via pipetting to ensure that no mycelium was removed and washed twice with fresh PDB/2 media. Stained mycelium was plated on a glass slide and imaged via confocal.

### Solid culture of fungal isolates

Once individual strains were isolated, they were grown on ME and PDB/2 agar plates where the boundary of the mycelium was outlined daily for a total of 7 to 10 days, depending on the strain. Approximately 30 ml was added to each petri dish (Thermo Fisher Scientific, cat#FB0875712, Waltham, MA, USA). Then each strain was cultivated on both plate types, outlining the edge of the mycelium daily. These were then scanned via Epson Perfection V39 II Scanner, and the average millimeter difference between daily bounds drawn was quantified via ImageJ (Version 1.54 h; [27]).

### Liquid culture of fungal isolates

In order to assess lipid content and the fatty acid profiles of these strains, they were first cultured in liquid PDB/2 media for five days. In order to assess lipid content and the fatty acid profiles of these strains, each fungal strain was cultured in liquid PDB/2 media for 5 days at 23C. A third of which was transferred to a new flask, and fresh media was added. After 14 days, the floating mycelium was collected and dried via freeze-drying with the Freezone 2.5 l Benchtop Freeze Dyer (Labconco, cat# 700201000, Kansas City, MO, USA). The freeze-dried mycelium was then stored at − 80° C until lipid extraction.

### Total lipid extraction and quantification

Lipid Extraction was performed by crushing the previously freeze-dried mycelium and transferring it to a pre-weighed screw gap glass tube, as reported previously [12]. 2 ml of chloroform: methanol: 88% formic acid (2:1:0.1) was added to the screw cap test tube and vortexed at 1500 RCF for 30 min with the Benchmixer XL Multi-tube Vortexer (Benchmark Scientific, cat# BV1010, Sayreville, NJ, USA). Then 0.5 ml of 1 M KCl and 0.2 M H_3_PO_4_ was added, and the phase was separated by centrifugation at 1000 RCF for 5 min. The lipid-containing precipitates were collected into pre-weighed screw cap glass tubes. These extraction steps were repeated 3 times, and lipid precipitates were combined and dried under N_2_ gas. These were then weighed to determine relative lipid content as a percentage of dry weight.

### Relative fatty acid quantification

After determining the lipid content, the relative abundance of fatty acids was determined via gas chromatography [12]. This was done by dissolving dried lipids in 1 ml of chloroform and transferring 100 µl to a new screw cap glass tube, and subsequently drying under nitrogen gas. Then, 100 µl of the internal standard (C13:0) and 1 ml of 1 M methanolic HCl were added, followed by incubation for 25 min at 80 °C, and cooled to room temperature. 1 ml of hexane and 1 ml of 0.9% NaCl were added and vortexed briefly. This was then centrifuged at 2500 RCF for 3 min, and the top hexane layer was transferred to a new test tube (Thermo Fisher Scientific, cat# 14-961-27, Waltham, MA, USA) and dried under N_2_ gas. Then this was resuspended in 120 µl of hexane and transferred to a 1.5-ml screw neck vial (Macherey–Nagel, cat# 702284, Düren, Germany) containing a 0.3-ml insert (Macherey–Nagel, cat# 702825, Düren, Germany). This was then loaded into a Gas Chromatography System (Aligent, Aligent 8860, Santa Clara, CA, USA), and the following protocol was executed: initial temperature 140 °C, increased by 25 °C/min to 160 °C, 8 °C/min to 250 °C, and then held at 250 °C for 15 min. 

### DNA extraction of fungal isolates

DNA extraction from mycelium was performed by obtaining a small piece of mycelium grown on PDB/2 plate with tweezers, no larger than 4 mm^2^, and placing it into a PCR tube with 20ul extraction solution (ES) [18]. ES was prepared with 10 ml of 1 M Tris, 1.86 g KCl, 0.37 g EDTA, and 80 ml deionized water, titrated with 1 M NaOH to pH 9.5–10. Then, an additional 100 ml deionized water was added, followed by filter sterilizing. The mycelium was then incubated in ES at room temperature for 10 min, followed by a 95 °C incubation on a C1000 touch thermal-cycler (BioRad, 1851196, Hercules, CA, USA) for 10 min. Afterward, 60 µl of dilution solution (DS) was added to the PCR tube. DS was prepared with 3 g BSA and 100 ml of deionized water, and the filter was sterilized. Extracted DNA was then stored at -20 °C until ready for PCR amplification.

### PCR application and sequencing of ITS and LSU sequence

In order to amplify the LSU rRNA gene, a master mix containing 1 ul of 10uM forward primer: LROR (ACCCGCTGAACTTAAGC), 1ul 10uM reverse primers: LR5 (TCCTGAGGGAAACTTCG) 25ul of OneTaq 2 × Master Mix with Standard Buffer (New England BioLabs, Cat. M0482S, Ipswich, MA, USA), 23ul of nuclease-free water, and 5ul of DNA template previously extracted. This was then amplified on a C1000 touch thermal-cycler (BioRad, 1851196, Hercules, CA, USA) using the following thermal-cycling conditions: 94 °C, 0:30; 94 °C, 0:20; 55 °C, 0:30; 68 °C, 1:12; Goto 2, 30x; 68 °C, 5:00; 4 °C, 0:00. The ITS rRNA gene was amplified using the primers ITS1F (CTTGGTCATTTAGAGGAAGTAA) and ITS4 (TCCTCCGCTTATTGATATGC) using the same PCR parameters, except with the primer annealing temperature of 53 °C. Amplified products were stored at – 20 °C until gel extraction.

The amplified LSU sequence was isolated via gel extraction where 40ul of the PCR products were run on 1% agarose gel for about 2-3H. The LSU band was then excised from the gel at ~ 1 KB based on a 1 KB ladder (New England BioLabs cat. N3232S, Ipswich, MA, USA). Excised bands were stored in a 1.5 ml tube at 4 °C until gel extraction could be performed following the manufacturer’s recommendations. The purified LSU sequence was stored at − 20 °C until Sanger sequencing could be performed.

Sanger sequencing was performed by the Advanced Studies in Genomic, Proteomics, and Bioinformatics at the University of Hawaii at Manoa where 100 ng of the purified template along with 3.2 ng of the LROR and LR5 primers was provided in order to receive forward and reverse sequences. Forward and reverse sequences were assembled into a consensus sequence utilizing the Generate Consensus Sequence function in the Geneious software [28].

### Phylogenetic tree assembly

LSU rDNA sequences generated in this study from environmental isolates were compared to similar sequences in the National Center for Biotechnology Information (NCBI) database with the BLASTn algorithm [29]. Sequences from isolates and reference sequences were aligned in Mesquite v3.81 [30] with the MUSCLE algorithm [31]. Gaps at the end of sequences were coded as missing data, and non-alignable regions were excluded from the analysis. Nucleotide substitution models and phylogeny were inferred through maximum likelihood with RAxML v2.0.10 based on 1000 ultrafast bootstrap replicates [32]. Phylogenetic trees were viewed in FigTree [33].

## Data Availability

No datasets were generated or analysed during the current study.
